# The Effect of Radioiodine on the Intima Media Thickness of the Carotid Artery

**DOI:** 10.4274/Mirt.24119

**Published:** 2013-12-10

**Authors:** Erdem Sürücü, Recep Bekiş, Tarık Şengöz, Yusuf Demir, Ahmet Orhan Çelik, Özge Orbay, Bilge Birlik, Özhan Ozdoğan, Enis İğci, Hatice Durak

**Affiliations:** 1 Yüzüncü Yıl University School of Medicine, Department of Nuclear Medicine, Van, Turkey; 2 Dokuz Eylül University School of Medicine, Department of Nuclear Medicine, İzmir, Turkey; 3 Dokuz Eylül University School of Medicine, Department of Radiology, Izmir, Turkey

**Keywords:** Hyperthyroidism, carotid intima-media thickness, iIodine radioisotopesy

## Abstract

**Aim:** The radiation can induce vessel injury. The result of this injury can be severe and life-threatening. There are a few studies demonstrating an increase in intima-media thickness (IMT) of the common carotid artery (CCA) after radiotherapy, especially in head and neck cancers. We evaluated the effect of I-131 to the IMT of the CCA in the patients who were treated for hyperthyroidism.

**Methods: **38 patients (25M, 13W) referred to our department for radioiodine treatment with the diagnosis of nodular goitre (25 patients) and diffuse hyperplasia (Graves disease (GD), 13 patients) were included to the prospective study. An USG was performed for all the patients before therapy, 3, 6 and 12 months after radioiodine therapy in order to measure IMT of CCA and the femoral artery (FA). The IMT was measured at the level of proximal part of bulbus anteriorly on the left and right side. The IMT of FA was measured just before the bifurcation.

**Results:** There was a statistically significant increase in IMT of both CCA and FA bilaterally in nodular hyperthyroid patients. However, in the patients with Graves disease, there was only statistically significant increase in the left IMT of CCA at 0-3rd, 0-6th month measurements and in the right IMT of FA at 0-3rd month measurements.

**Conclusion:** Though the limitation of the study is the interobserver and intraobserver variability, it was seen that I-131 therapy might affect the IMT of CCA in the patients with NG. I-131 effect on the IMT of CCA in patients with nodular goitre was higher than the IMT of CCA in patients with GD. I-131 effect on the IMT of CCA might be due to administered dose and adjacency. The interesting point of our study was the increased thickness of IMT in FA. We think that the increase in IMT is due to the systemic effect of radioactivity circulating in the blood vessel. I-131 effect on the IMT of FA in patients with nodular goitre was higher than the IMT of FA in the patients with GD due to I-131 uptake of thyroid gland. Because I-131 uptake was lower in patients with nodular goitre, I-131 in systemic circulation was higher.

**Conflict of interest:**None declared.

## INTRODUCTION

It was shown that therapeutic radiation exposure cause damage to the carotid and other large vessels in the world literature since the 1950s (1,2,3). The characteristics of damage and pathological changes for atherosclerosis were demonstrated in the animals (4,5). In the literature, acute thrombosis, carotid rupture, carotid wall thickening and rapidly progressive atherosclerosis were reported due to radiation exposure in the carotid vessels (1,2,3,6,7). A higher incidence of significant extracranial carotid artery stenosis was shown in patients who received external irradiation to the head and neck area for any kind of malignancy.

Radioiodine therapy is a useful option for the treatment of hyperthyroidism with antithyroid drugs (ATDs) and surgery. Iodine-131 penetrates to the tissue due to beta radiation resulting in cell death. Radioiodine is in most cases the first-line treatment for solitary hyperfunctioning thyroid nodules. It is also used in uncontrolled hyperthyroidism and Graves’ disease. Our hypothesis is beta radiation can cause damage to the adjacent vessels of thyroid lobe while killing hyperfunctioning thyroid cells. We investigated the effect of radioiodine on the intima media thickness (IMT) of the carotid artery. This is the first prospective study to evaluate the effects of radioiodine on the carotid artery wall in the literature.

## MATERIALS AND METHODS

The research protocol was approved by the local ethics committee of clinical and laboratory research in Dokuz Eylül University School of Medicine and informed consents were obtained from all patients.

**Patients**

All patients with nodular goitre and diffuse hyperplasia which were referred to our clinic for I-131 therapy were included in the study between July 2009 and January 2012. The patients with diabetes, smoking, high blood pressure and high blood cholesterol that can affect the vascular tissue were excluded.

38 patients (25M, 13W) were included in the study. 25 of 38 patients were diagnosed as nodular goitre (NG), 13 of 38 patients were diagnosed as diffuse hyperplasia (Graves disease (GD)). The average given dosage of Iodine 131 was 14.7 mCi (10-20 mCi). 36 patients were followed up to 12 months in order to evaluate the therapy efficiency. Two patients moved to another city, so, 12th month control USGs were not performed. 4th and 24th hour I-131 uptakes were measured for all patients before I-131 therapy.

**Intima-media thickness**

**Figure 1:**
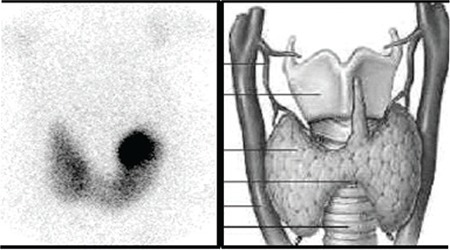
The adjacency between the carotid artery, thyroid gland andthe possible accumulation of I-131 in a nodule (hyperactive nodule) wasshown (Scintigraphic image with Tc 99m pertechnetate was obtained fromthe Department of Nuclear Medicine in Dokuz Eylül University, School ofMedicine and picture in gray scale was obtained from the web site “http://www.saglik.im/hipotiroidizm/”)

The inner wall is usually expressed as the IMT. The IMT was defined as the distance between the echogenic line representing the blood-intima interface and the echogenic line representing the media adventitia interface. An USG was performed for all the patients before the radioiodine therapy for basal IMT (0) and 3, 6, 12 months after radioiodine therapy in order to observe any increment of IMT of CCA and the femoral artery (FA). USG was performed by two radiologists who had five years of experience in USG and they did not know which patient had NG or GD and which month is being measured. All scans were obtained with Philips ATL5000 (Holland, Eindhoven), by using a 7.5 MHz linear array transducer and standardized machine settings in a preset carotid arterial and low extremity arterial imaging program. Patients were examined in the supine position. The IMT was measured at the level of proximal part of bulbus anteriorly on the left and right side for CCA (Figure 1) and just before the bifurcation for FA with the transducer head perpendicular to the vessels. Measurements of the IMT were performed on magnified static images with electronic callipers. Measurements were recorded and averaged. A description of the plaques was noted in the study.

**Statistical analysis**

SPSS for windows 11.0 program (SPSS® 11, Chicago, IL, USA) was used for statistical evaluation and descriptive analysis. Significant difference between 0-3, 0-6, 0-12 months were evaluated with Friedman Test, Wilcoxon Signed test, correlation between dosage of I-131 and increments of IMT, 4th-24th hour I-131 uptake and increments of IMT were evaluated with Spearman’s rho test with a statistical significance chosen at p<0.05.

## RESULTS

**Patients**

38 patients (25M, 13W) were evaluated for the IMT of carotid and femoral artery. 25 of 38 (66%) patients had NG and 13 of 38 (34%) patients had GD. 12 of 25 patients had nodule in the left thyroid lobe, 9 of 25 patients had nodule in the right thyroid lobe and 4 of 25 patients had nodules in bilateral thyroid lobes. GD patients were treated with fixed doses of 10 mCi, NG patients were treated with 15-20 mCi Iodine-131. Patient characteristics are summarized in Table 1.

Intima-media thickness

The average IMT of the carotid and femoral arteries are shown in Table 2. The IMT of right carotid artery increased from 0.84±0.20 to 0.90±0.20 (p=0.028); the IMT of left carotid artery increased from 0.88±0.22 to 0.94±0.23 (p=0.033); the IMT of right femoral artery increased from 0.96±0.24 to 1.06±0.26 (p=0.001); the IMT of left femoral artery increased from 0.97±0.26 to 1.07±0.25 (p=0.009) in patients with NG at the end of 12th month. Statistical analysis on the increment of IMT between 0-3rd, 0-6th, 0-12th months are shown in Table 2. It can be seen that there is a significant increase in the IMT of carotid and femoral arteries between 3rd, 6th, 12th months and baseline measurements in patients with NG. No significant difference was seen between the dosage of I-131 and increments of IMT in all arteries and between the 4th-24th hour I-131 uptake and increments of IMT in all arteries for both groups.

## DISCUSSION

Our study demonstrates blood vessel injury in response to I-131 treatment of hyperthyroidism. There is no study about the effect of radioiodine on IMT of CCA in the literature. Our study showed that the radioiodine affect the IMT of the arteries. Changes in the artery after radiation exposure to the carotid artery are similar to age-related atherosclerosis (8). It has been demonstrated that radiotherapy induces acute endothelial injury. Animal studies have shown fibrosis in the carotid arteries that was secondary to radiotherapy in relationship with irradiation dose and time (9). Histopathologic changes cause atherosclerotic-like plaques resulting in vascular stenosis and thromboembolic processes (10). Endothelial damage causes endothelial proliferation (11,12), chronic fibrosis of the intima media and occlusive changes in the vasa vasorum of the adventitia that result in vascular stenosis processes (10).

Our study is the first prospective study that the effect of radioiodine is investigated in the literature. It demonstrates that radioiodine effects IMT of the carotid and femoral arteries. This effect can be a local effect to the CCA and/or might be a systemic effect to the FA, because significant increases were seen in IMT of both CCA and FA in the patients with NG. High dose of I-131 (mean 17.4 mCi) was given to the patients with NG while fixed dose (10 mCi) of I-131 was given to the patients with GD. So, the statistical difference in the CCA between the patients with NG and GD can be due to the amount of I-131 dose. 4th and 24th hour uptakes were higher in patients with GD than the patients with NG. So, the amount of iodine was higher in systemic circulation in patients with NG. Systemic effect of I-131 might be due to high iodine in systemic circulation and also the amount of iodine might cause the statistical difference in FA in the patients with NG.

O’Leary and et al. (13) showed that an increment of 0.55 mm in wall thickness had an approximate 40% increased risk of stroke. The increase of IMT in any patient can be a potential risk factor that causes stroke. However, in our study the highest increment was 0.1 mm. So, this increment may not be important clinically.

The limitation of the study was the interobserver and intraobserver variability. In the literature, interobserver and intraobserver variability in the selected studies were reported between a mean difference of 0.02-0.66 mm and 0.01 and 0.65 (14). The measurements for IMT are so small (mm) and the given I-131 dose is low. The interobserver and intraobserver variability might affect the results and the statistical difference might be obtained because of these variabilities, however, the statistical difference has occurred in all arteries in patients with NG.

On the other hand, age might also be a pitfall of the study. The average ages were different in both groups (60.8±14.7 and 51.2±16.2). The statistical difference in patients with NG might be secondary to the older ages. However, in a study with 2265 patients, it was demonstrated that IMT increased 5.7 +/- 0.4 μm/year. In our study, the IMT of right carotid artery increased from 0.84±0.20 to 0.90±0.20 (0.06 mm=60 μm); the IMT of left carotid artery increased from 0.88±0.22 to 0.94±0.23 (0.06 mm=60 μm), the IMT of right femoral artery increased from 0.96±0.24 to 1.06±0.26 (0.1 mm=100 μm); the IMT of left femoral artery increased from 0.97±0.26 to 1.07±0.25 (0.1mm=100 μm) in NG group at the end of 12th month. So, the increments in our study are higher than the age-related increments. Thus, we thought that this effect can be due to radiation (15).

It has been reported that radiation-related changes take 10 to 15 years to reach clinical significance (8). However, animal studies demonstrated the effect of radiation in the carotid artery within days of radiation exposure (4,5). Mean thickness of the common carotid artery increases with age at a rate of 0.008 mm/year (16). In a prospective study involving 36 patients, Muzaffar et al. (8) demonstrated that external irradiation significantly increases the carotid IMT during the first year after irradiation. There is no statistical difference between 6th and 12th months in IMT of CA for the patients with NG. While the average right IMT of the patients with NG is 0.91±0.20 in 6th month, it is 0.90±0.20 in 12th month. While the average left IMT of the patients with NG is 0.96±0.22 in 6th month, it is 0.94±0.23 in 12th month. According to our study, it was seen that the iodine effects until the 6th month.

In conclusion, it appears that radioiodine effects the IMT of carotid artery locally and FA systematically. Furthermore, longitudinal prospective studies are needed to analyse the local and systemic IMT-changes in large number of patients that were treated with iodine-131 or in patients with papillary thyroid cancer who received high dose of iodine-131 in ablation therapy.

## Figures and Tables

**Table 1:**
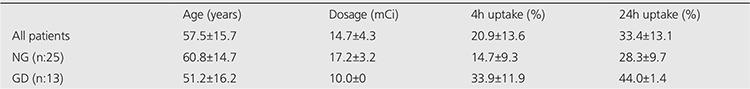
Patient characteristics

**Table 2:**
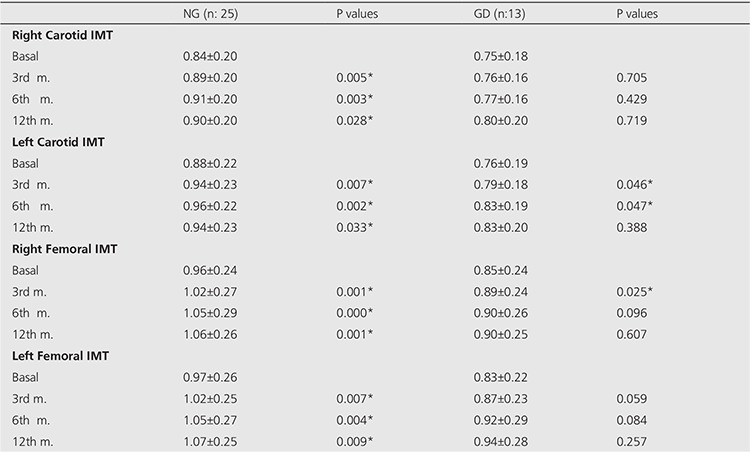
The average IMT of the carotid and femoral arteries (mm)
